# Supplementary material for: High Expression Levels of miR-21-5p in Younger Hospitalized COVID-19 Patients Are Associated with Mortality and Critical Disease
Source: Int J Mol Sci. 2023 Jun 14;24(12):10112. doi: 10.3390/ijms241210112 (PMC10298718; doi:10.3390/ijms241210112)
Supplement: Supplementary file 1 [file ijms-24-10112-s001.zip › ijms-2418292-supplementary.pdf]

Supplementary Material

1 Supplementary Data

Supplementary Table S1: Characteristics of miR-25-5p and miR-146a-5p.

Characteristics	miR-21-5p	miR-146a-5p
Localization	Chromosome 17, at cytogenetic band 17q23.1	Chromosome 5, at cytogenetic band 5q33.1
length of sequence	22 base pairs	22 base pairs
Mature miRNA Sequence	UAGCUUAUCAGACUGAUGUUGA	UGAGAACUGAAUUCCAUGGGUU
Genes target related to the pathophysiology of COVID-19.	• FAS Ligand<br>• Transforming growth factor-beta.<br>• Interleukin 12<br>• Programmed cell death protein 4<br>• Mitogen-Activated Protein Kinase Kinase Kinase 1<br>• Fibroblast growth factor	• TNF Receptor Associated Factor 6<br>• Receptor-associated kinase 1<br>• Platelet-derived growth factor alpha receptor<br>• Chemokine receptor 9 with cc motif<br>• Family of SMAD

Are listed some target genes of both miRNAs related to COVID-19.

Supplementary Table S2: Cox regression analysis for the multivariable assessment of mortality.

**Characteristics**	**Hazard ratio**	***p*-value**	**95% CI**
Age, years	1.032	0.150	0.989 - 1.077
2–ΔCt-miR-21-5p	1.096	0.418	0.878 – 1.367
2–ΔCt-miR-146a-5p	1.065	0.551	0.866 – 1.309

Cox regression analysis for the multivariable assessment of mortality, Omnibus test of model coefficients p = 0.288.

Supplementary Table S3: miR-21-5p and miR-146a-5p and their relation with comorbidities in patients with severe and critical disease in COVID-19.

**Variable**	**miR-21-5p**		**miR-146a-5p**	
	FC	*p*	FC	*p*
**T2DM**	0.249 in favor of the patients with T2MD.	0.70	0.500 in favor of the patients with T2DM.	0.42
**SAH**	0.028 in favor of the group with SAH.	0.95	0.958 in favor of the group with SAH.	0.45
**Tobacco smoking**	0.166 in favor of the tobacco-smoking subjects.	0.85	0.520 in favor of the tobacco-smoking subjects.	0.22
**Obesity**	0.001 in favor of the group with obesity.	0.93	0.165 in favor of the group with obesity.	0.37

U Mann-Whitney statistical test was employed for the comparisons between groups. Obesity (BMI > 30); SAH, systemic arterial hypertension; T2DM, Type 2 Diabetes Mellitus.

Supplementary Table S4: Clinical and laboratory variables included in the correlation tests

Red blood cells (erythrocytes), Hemoglobin, Hematocrit, and other variables. White blood cells: Leukocytes, Neutrophils, Lymphocytes, Monocytes, Macrophages, Eosinophils, Basophils, Prothrombin time, Activated partial thromboplastin time, INR, Platelets, D-Dimer, Ferritin, C protein reactive, Procalcitonin. Liver function: Alanine transaminase (ALT), Aspartate transaminase (AST), Alkaline phosphatase (ALP), Albumin, Bilirubin direct, indirect, and total. Kidney function: Urea, BUN, Creatinine, Glucose. Electrolytes: Potassium, Sodium, Calcium, Magnesium, Phosphorus. Blood fat: Total cholesterol (TC), HDL cholesterol, LDL cholesterol, a TC/HDL ratio, and Triglycerides.

Supplementary figures

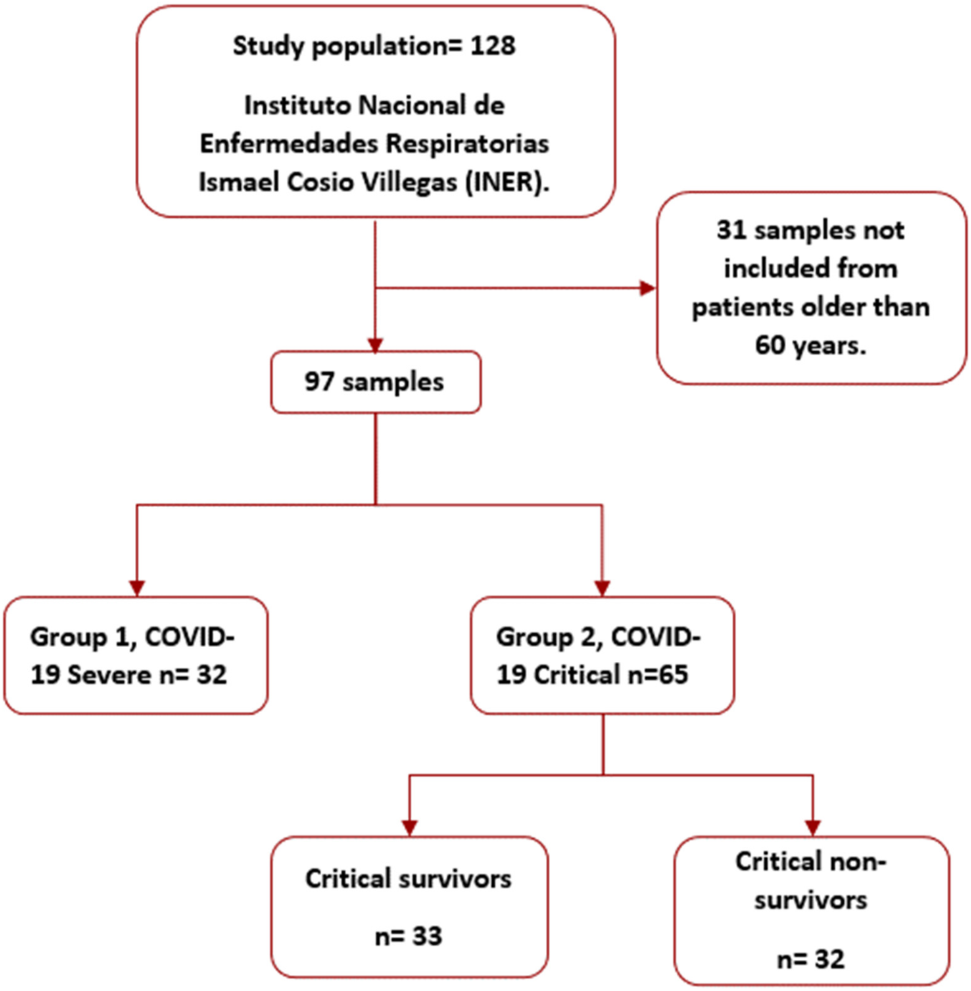

Supplementary Figure S1: Stratification of study population.

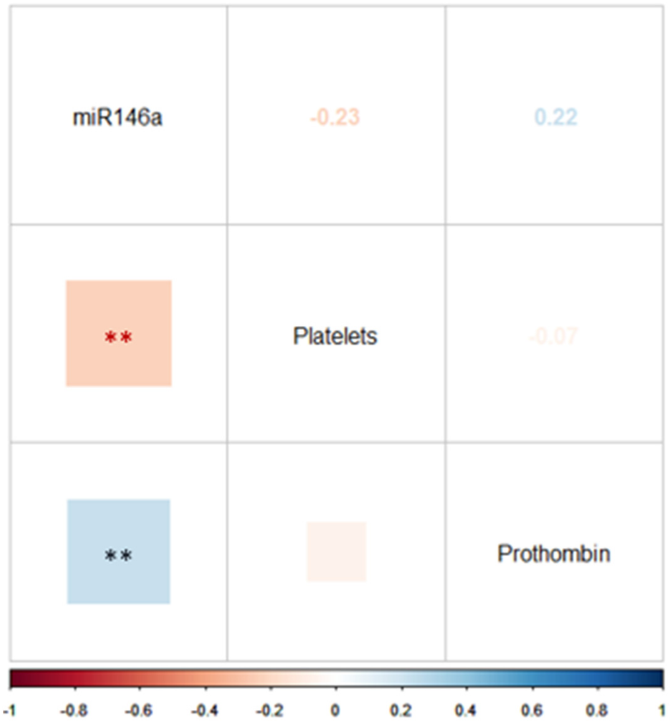

Supplementary Figure S2: Statistically significant correlations between miR-146a-5p and clinical variables. ** $p < 0.001$.
